# Moving the needle on dental antibiotic overuse in Canada post COVID-19

**DOI:** 10.14745/ccdr.v48i1112a02

**Published:** 2022-11-03

**Authors:** Susan Sutherland, Karen Born, Sonica Singhal

**Affiliations:** 1 Sunnybrook Health Sciences Centre, Toronto, ON; 2 Faculty of Dentistry, University of Toronto, Toronto, ON; 3 Institute of Health Policy, Management, and Evaluation, University of Toronto, Toronto, ON; 4 Health Promotion, Chronic Disease, and Injury Prevention, Public Health Ontario, Toronto, ON

**Keywords:** dental, COVID-19, antibiotic overuse, antimicrobial resistance, antimicrobial stewardship

## Abstract

Antimicrobial resistance due to over-prescribing in health care, including in dentistry, has been acknowledged as one of the top ten threats to global health by the World Health Organization. Dentistry is responsible for approximately 10% of antibiotics prescribed worldwide and research has shown up to 80% of antibiotics prescribed by dentists may be unnecessary. During the early months of the coronavirus disease 2019 pandemic, when dental offices handled only dental emergencies, it is probable that antibiotics were prescribed more readily and for longer duration to defer treatment for non-urgent cases. These unprecedented times strengthened the realization that strong dental antimicrobial stewardship practises are required in Canada to keep antimicrobial overuse under control. In countries, such as the United Kingdom and Australia, significant work is ongoing in this regard. Canada has made progress in developing tools for antimicrobial stewardship specifically for physicians in community settings, where the vast majority of antibiotics are prescribed, and it is now time to pay attention to antimicrobial stewardship in the field of dental care. Investments in developing a national level dental prescription database, along with monitoring, education and feedback mechanisms, can strongly support moving the needle on dentist-driven antibiotic overuse in Canada.

## Introduction

At the G7 Health Ministers meeting in Berlin in May 2022, antimicrobial resistance was listed as one of four priority areas of focus, along with coronavirus disease 2019 (COVID-19), future pandemic preparedness and health risks from climate change (1). Antibiotics are essential to the practice of dentistry for the prevention of distant site infections such as infectious endocarditis, as adjuncts for the prevention of some surgical site infections, and for the treatment of serious odontogenic infections.

Globally, dentistry is responsible for approximately 10% of antibiotics prescribed across health care, and research has shown up to 80% of dental antibiotics may be unnecessary, with wide variation between countries (2). Reliable information on prescribing by dentists is not available in most Canadian provinces, but data from the BC PharmaNet database indicates that during a ten-year period, antibiotic prescriptions by physicians decreased by 18.2%, while prescriptions by dentists increased by 62.2% (3). The reasons for this are unclear, but self-reported data from a 2016 survey of Canadian dentists (4) indicated that there is misunderstanding by dentists of both the medical indications as well as the dental procedures requiring antibiotic prophylaxis for the prevention of infective endocarditis. A lack of awareness of changes to antibiotic guidelines for patients with total joint replacement, variation in prescribing practices among dentists for antibiotic prophylaxis for the prevention of surgical site infections, use of antibiotics for conditions where antibiotics are not necessary and general overuse of clindamycin and underuse of penicillin V. Furthermore, where the most appropriate management of dental infections (surgical intervention) is most likely unavailable, visits to family physicians and emergency departments for non-traumatic dental conditions (5), may result in inappropriate antibiotic prescribing.

The COVID-19 pandemic has had a profound impact on oral health and dental practices worldwide. Deferred care during the early months of the pandemic created a huge backlog of needed dental treatment. During the months of virtual triage or office closures, with only the most urgent care provided in person, it is not difficult to imagine that when patients presented with dental issues, antibiotics were prescribed more readily and for longer duration. Data from the United Kingdom and Alberta support this (6,7). The contribution of dentist-driven prescription adds to global increase in antibiotic prescribing across health care as a result of the pandemic (8).

## Dental antimicrobial stewardship

The World Dental Federation encourages all national dental associations across all low, middle and high-income economies to commit to antimicrobial stewardship (AMS) by advocating for the inclusion of dentistry in national action plans and supporting their members to prescribe antibiotics wisely (9,10). To date, 58 national dental associations, including the Canadian Dental Association, have taken the World Dental Federation Pledge to tackle antibiotic resistance and enhance patient safety in their countries through three pillars: raising awareness and understanding about the concerns associated through effective communication, education and training; reducing the incidence of dental infection through effective sanitation, hygiene and infection prevention and control measures; and optimizing the use of antibiotics in human health.

Dental AMS programs focus primarily on reducing inappropriate antibiotic prescribing. Whilst reducing the use of antibiotics in dentistry is important, the significance of changes in prescribing rates to patient outcomes is poorly understood (11). It is important to ascertain that harm to patients is also reduced through the study of patient-related clinical outcomes (11). From a behavioural sciences perspective, it can be challenging to convince patients and clinicians to avoid an unnecessary antibiotic prescription due to its contribution to antimicrobial resistance. Describing the individual risks and benefits of an unnecessary antibiotic prescription can help support shared decision-making to avoid unnecessary antibiotic prescribing (12). To this end, research is underway to develop an international consensus on a core outcome set for dental AMS (13). At the present time, however, efforts comprise a combination of dissemination of guidelines, educational components for both clinicians and patients, and audit and feedback to improve dental antibiotic prescribing.

Guidelines for appropriate dental antibiotic use have largely focused on prophylaxis of distant site infections such as infective endocarditis and late prosthetic joint infections. These vary significantly by region and there continues to be some controversy (14). In Canada, the American Heart Association guidelines are followed for prevention of infective endocarditis (15) and the tripartite consensus statement from the Canadian Dental Association, the Canadian Orthopaedic Association and the Association of Medical Microbiology and Infectious Disease Canada provides solid advice against the use of antibiotics for patients with total joint replacement (16). Useful guidelines have recently been published by the American Dental Association on the use of antibiotics in the management of dental pain and/or intra-oral swelling (17).

Educational interventions for dentists are increasingly focused on toolkits, designed using concepts from the behavioural change literature and co-designed with patients. Significant work is ongoing in the United Kingdom and Australia in this regard (18-21). Although specific to care delivery in those countries, many of the concepts and tools can be adapted for Canadian dental practice. Similarly, through its Using Antibiotics Wisely campaign, Choosing Wisely Canada has developed excellent tools for physicians and resources for patients, which include a “viral prescription pad” and delayed prescription, alongside educational posters and pamphlets (22). Opportunities exist to leverage this work in the development of dental AMS strategies.

“Audit and feedback” is used to measure an individual’s professional practice, compare it to targets, professional standards or peer performance, and provide feedback to the individual to improve quality of care. It can lead to small but potentially important improvements in professional practice, especially when baseline performance is low, and the feedback is carefully designed and delivered (23). This method has been shown to improve antibiotic prescribing in Canadian medical practice (24) and dental antibiotic prescribing in Scotland (25). Future studies to assess the most impactful design approach to audit and feedback are being planned in medicine (26) and dentistry (27). That said, because dental care is privately funded and delivered in Canada, accessibility to dental prescribing data is a challenge.

## How can we move the needle forward in Canadian dentistry?

Canadian efforts in dental AMS are nascent, but there is strong interest and support for moving forward (28). The Canadian dental profession is well positioned to evaluate international dental AMS programs, as well as programs developed in medicine such as Choosing Wisely campaigns, to develop a strategy for a Canadian dental AMS program. Learning from international experiences in the field of dentistry can provide opportunities to implement such strategies in the Canadian context. To help move the needle forward, the authors have received a research grant from the tri-university Manchester-Melbourne-Toronto (MMT) Research Fund June 2022 competition. The funding is for the specific purpose of holding a workshop in Toronto in the fall of 2023, the goal of which will be to develop a strategic framework and action plan for AMS in Canadian dentistry, with international contributions from experienced researchers in the field from Manchester and Melbourne. Engagement with key dental and inter-professional stakeholders and organizations, as well as patients and members of the public, will help to shape this initiative and, we hope, provide momentum for change.

## Conclusion

Addressing the significant data gap in dental antibiotic prescribing will be challenging. The likely implementation of the National Dental Care Program, targeting more than six million Canadians, presents an opportunity for establishing a dental prescription database at the national level, which can be routinely monitored to support reviewing the prescription practises of participating dentists across provinces and territories (29). Lessons learned emerging from the workshop may suggest other processes to explore in this regard. This will also support developing audit processes and feedback strategies to ultimately move the needle forward to optimize antibiotic prescribing practices among Canadian dentists.
